# Wee1 epigenetically modulates H2B mono‐ubiquitination at K120 lysine and DNA double‐strand break repair through phosphorylation of H2BY37‐dependent manner in small‐cell lung cancer

**DOI:** 10.1111/1759-7714.14862

**Published:** 2023-04-26

**Authors:** Xiaoliang Zhao, Xiaohua Wen, Bin Liu

**Affiliations:** ^1^ Department of Lung Cancer, Tianjin Key Laboratory of Cancer Prevention and Therapy, National Clinical Research Center for Cancer Tianjin Medical University Cancer Institute and Hospital Tianjin China; ^2^ Tianjin University of Traditional Chinese Medicine Tianjin China; ^3^ Office of Academic Research Affiliated Hospital of Hebei University Baoding China

**Keywords:** DNA double‐strand break (DSB) repair, H2Bub, small cell lung cancer (SCLC), Wee1

## Abstract

**Background:**

DNA damage repair is a crucial mechanism highly related to therapy resistance for various therapeutic strategies. Our previous results have shown that the degree of drug resistance in small‐cell lung cancer (SCLC) cell lines was proportional to both the transcription and expression levels of Wee1, indicating that Wee1, an evolutionarily highly conserved kinase, plays a vital role in the therapeutic resistance of SCLC. In the present study, we aim to determine the nonclassical mechanism of Wee1 on DNA repair regulation.

**Methods:**

Western blot was conducted to determine the mono‐ubiquitination level of H2Bub. Comet assay was used to evaluate the degree of DNA damage. Immunofluorescence was conducted to determine the DNA repair markers. Co‐immunoprecipitation was utilized to assess the potential interactions with H2BY37ph. MTT assays were used to evaluate the survival rates of SCLC cells.

**Results:**

Overexpression of Wee1 increases the level of H2BK120ub and alleviates ionizing radiation (IR)‐induced DNA damage in SCLC cells. Moreover, H2BK120ub is a crucial molecule in Wee1‐mediated double‐strain break (DSB) repair in SCLC cells. Mechanisms study indicated that H2BY37ph is involved in Wee1‐mediated H2BK120ub through interaction with the E3 ubiquitin ligase RNF20–RNF40 complex and upregulates its phosphorylation, mutation of H2BY37 phosphorylation sites attenuated DSB repair and enhanced the sensitivity of IR‐induced SCLC cell death.

**Conclusion:**

H2BY37ph produces crosstalk with H2BK120ub in an E3 ubiquitin ligase‐dependent manner, promoting Wee1‐mediated DSB repair in SCLC cells. This study clarifies the nonclassical mechanism of Wee1 regulation of DSB repair, which provides a theoretical basis for the clinical understanding of the regulatory network of Wee1 and its use as a target for overcoming multiple types of therapeutic resistance.

## Graphical abstract

Wee1 epigenetically modulates H2B mono‐ubiquitination at K120 lysine and DNA double‐strand break (DSB) repair through phosphorylation in an H2BY37‐dependent manner in small‐cell lung cancer (SCLC). Overexpression of Wee1 increases the level of H2BK120ub and alleviates ionizing radiation (IR)‐induced DNA damage in SCLC cells. H2BK120ub is a crucial molecule in Wee1‐mediated DSB repair in SCLC cells. H2BY37ph is involved in Wee1‐mediated H2BK120ub through interaction with the E3 ubiquitin ligase RNF20‐RNF40 complex and upregulates its phosphorylation, mutation of H2BY37 phosphorylation sites attenuated DSB repair and enhanced the sensitivity of IR‐induced SCLC cell death.

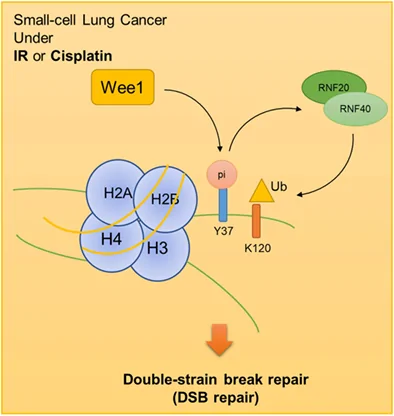

## INTRODUCTION

Lung cancer is a highly lethal malignant disease worldwide, particularly in China, where it has replaced hepatocellular carcinoma as the leading cause of death among all malignancies since 2008.^1^ As a more aggressive subtype, although small‐cell lung cancer (SCLC) accounts for only about 15% of all lung cancer diagnoses, its 5‐year survival rate is less than 7%.^2^ More regrettably, unlike epidermal growth factor receptor and other treatment options for managing non‐small‐cell lung cancer (NSCLC), there is no clinically proven target for SCLC treatment.^3^ Hence, novel management approaches for SCLC urgently need to be developed.

One of the representative characteristics of SCLC is the high mutation burden, which causes genomic instability—a hallmark of SCLC—resulting in rapid proliferation and replication stress in SCLC.^4^ Moreover, much research has verified the overexpression of DNA damage repair (DDR) proteins, such as Wee1, in SCLC tumor tissues compared to paracancerous.^5^, ^6^ In addition, one of the most potent activators of DDR is DNA double‐strand break (DSB), which is always induced by cisplatin and ionizing radiation (IR), therefore targeting the DDR pathway is a potential therapeutic strategy for SCLC and a potential combination approach with radiotherapy.^7^

As an evolutionarily conserved nuclear tyrosine kinase, Wee1 is an essential component of the G2/M cell cycle checkpoint, preventing mitotic entry under the condition of DNA damage. In addition, Wee1 also acts as a hub for regulating chromatin integrity. Wee1 could mark the completion of DNA replication and genetic integrity via Y15 phosphorylation and H2B Y37 phosphorylation, inhibiting Wee1 function and inducing mitotic catastrophe, therefore Wee1 is a crucial regulator for DNA replication, histone transcription, and chromosome condensation, making it a potential target for cancer treatment, especially for combination use with a DSB conductor (e.g., radio‐sensitizing effects).

Histone posttranslational modifications (PTMs) are crucial biological access to control DDR. More importantly, mono‐ubiquitination on lysine 120 of H2B (H2BK120ub) and its de‐ubiquitination have been reported to involve DDR as a critical mechanism of action. In yeast and humans, H2Bub regulation is a conserved process catalyzed by the E3 ubiquitin‐conjugating enzyme RNF20/40 complex and deubiquitinated by the mammalian SAGA complex. DSB‐inducing method, IR, induces a global increase in H2Bub levels. Moreover, the ubiquitylation of histone H2B also facilitates the replicative bypass of fork‐stalling DNA lesions by contributing to both DNA damage tolerance and homologous recombination during and after replication. However, more is needed for H2Bub on Wee1‐mediated DDR under DSB. The present study illustrates that H2Bub at lysine 120 is a crucial modification of Wee1‐mediated DDR by IR‐induced DSB in SCLC cells. Moreover, as a substrate of Wee1, phosphorylation of H2B at tyrosine (H2B Y37) is essential for Wee1‐induced H2Bub under IR in an RNF20/40‐dependent manner. Our findings make a further explanation on Wee1‐mediated DDR.

In the present study, we aim to determine the novel molecular mechanisms of action on Wee1‐mediated DSB repair through the crosstalk of H2BY37ph with H2BK120ub.

## MATERIALS AND METHODS

### Cell culture and reagents

Human SCLC cell line DMS114 was purchased from Cobio Biosciences Co,. Ltd. Cells were cultured in RPMI1640 medium containing 10% fetal bovine serum and maintained in a humid clean atmosphere with 5% CO_2_ at 37°C. Cells were cultured in tissue culture plates to 70% confluence 24 h prior to polyethyleneimine transfection with lentiviral packaging plasmids and the Wee1, H2B WT, H2BK12OR, and H2BY37F plasmid. Lentiviral culture supernatants were harvested at 2–3 days posttransfection and overlaid on target cells in a medium containing 10 μg/mL Polybrene. Transduced cells were selected at 24 h posttransduction in a complete medium containing 1 μg/mL puromycin or 2 mg/mL hygromycin for 3–6 days. X‐ray irradiation at 6 MV (600CD; Varian) at a dose rate of 2 Gy/min was used.

### Comet assay

DMS114 cells were collected with a density of 5 × 10^5^/mL, and 10 μL of diluted cells was collected in 0.5% low melting point agarose. After gelling at 4°C, the slides were then immersed in lysis solution for 1 h. Following lysis, the slides were washed three times with enzyme buffer. After buffer change, the slides were covered with formamido pyrimidine glycosylase in the enzyme buffer, sealed with a coverslip, and incubated in a humidified chamber for 45 min at 37°C. The slides were immersed in a cold alkaline electrophoresis buffer for 30 min to unwind the DNA. Electrophoresis was then performed at 25 V, 300 mA for 20 min. Slides were washed in neutralization buffer for 10 min followed by 100% ethanol for 5 min at room temperature and air‐dried. Finally, slides were stained with 5 μg/mL propidium iodide and imaged on a fluorescent microscope (Olympus). The average olive tail moment was analyzed (50 cells/slide) using Comet Assay Software Project Casp‐1.2.2 (University of Wroclaw, Poland). All experiments were repeated three times.

### Western blot

Cells were washed with iced PBS and collected, centrifuged, and resuspended by RIPA lysis buffer containing PMSF as well as phosphatase inhibitors. The cell lysis was incubated on ice for 30 min, then centrifuged at 14 000*g* for 15 min. The supernatant was collected, and the Bicinchoninic acid method was used to determine the quantity of the protein. After normalizing the protein quantity of the samples, a total of 20 μg of proteins was subjected to SDS‐PAGE and then transferred onto a PVDF membrane (Merck Millipore). BSA (5%) was used to block the nonspecifical binding. Primary antibodies were incubated at 4°C overnight, then the membranes were washed with Tris Buffered Saline with Tween‐20 (TBST) buffer and incubated with relative secondary antibodies for 2 h at room temperature. After being washed with TBST, the protein bands were visualized by enhanced chemiluminescence (ThermoFisher Scientific). The signals were captured by the photographic film in a dark room or by the BIO‐RADXR system (Bio‐Rad).

### Immunofluorescence

Cells were seeded on sterile glass coverslips, placed in six‐well plates, fixed with 4% paraformaldehyde, and permeabilized with 0.2% Triton X‐100. The cells were washed with PBS and blocked with 5% goat serum. Primary antibodies were incubated at 4°C overnight with specific dilutions. The results were captured by an FV3000 confocal microscope (Olympus) and analyzed by Image J software (Rawak Software Inc.).

### Co‐immunoprecipitation

Cells were harvested, added to an appropriate amount of cell lysis buffer (containing protease inhibitor), and lysed on ice for 30 min. The cell lysate was centrifuged at 4°C for 30 min at maximum speed and the supernatant was removed. Then, a small amount of lysate was taken for Western blot analysis, and the remaining lysate plus the corresponding antibody was added to the cell lysate and incubated overnight at 4°C with slow shaking. Protein A agarose beads were washed three times with appropriate lysis buffer, centrifuged at 3000 rpm for 3 min each time, then added to the cell lysate incubated overnight with the antibody and incubated for 2 h at 4°C with slow shaking to couple the antibody to the beads. After the immunoprecipitation reaction, the beads were centrifuged at 4°C at 2500 *g* for 3 min. The supernatant was aspirated and the beads were washed three times with 1 mL of lysis buffer and analyzed by SDS‐PAGE.

### Cell viability

A CCK‐8 kit was used to evaluate the proliferation of SCLC cells. First, 5 × 10^3^ cells/well were cultured in 96‐well plates in 180 μL of complete medium and treated with IR. After incubation for 24 h, the leaves were centrifuged and the medium was discarded. Subsequently, the cells were incubated with a complete medium (100 μL) containing CCK‐8 (10 μL) for 4 h at 37°C. An iMark multi‐plate reader (Bio‐Rad) was conducted to obtain each well's optical density (OD) (under 450 nm) value. The following equation was used to calculate the relative cell viability: cell viability = (OD_representing group_ − OD_blank_)/(OD_control group_ − OD_blank_) × 100%. The median inhibitory concentration (IC_50_) was calculated by Graphpad 9.00 Software using the nonlinear regression (curve fit) model.

### Statistics

The data were analyzed by Graphpad 9.00 software. The data were expressed as mean ± SD and three independent experiments were conducted. An unpaired Student's *t*‐test was used to  evaluate the significant differences between the two groups. Multigroup comparisons of the means were carried out by a one‐way analysis of variance (ANOVA) test with post hoc contrasts by Student–Newman–Keuls tests when the comparing group number no more than three. For groups of more than three, Tukey's test was conducted post hoc. The statistical significance for all tests was set when *p* was less than 0.05.

## RESULTS

### Overexpression of Wee1 increases the level of H2BK120ub and alleviates IR‐induced DNA damage in SCLC cells

**FIGURE 1:**
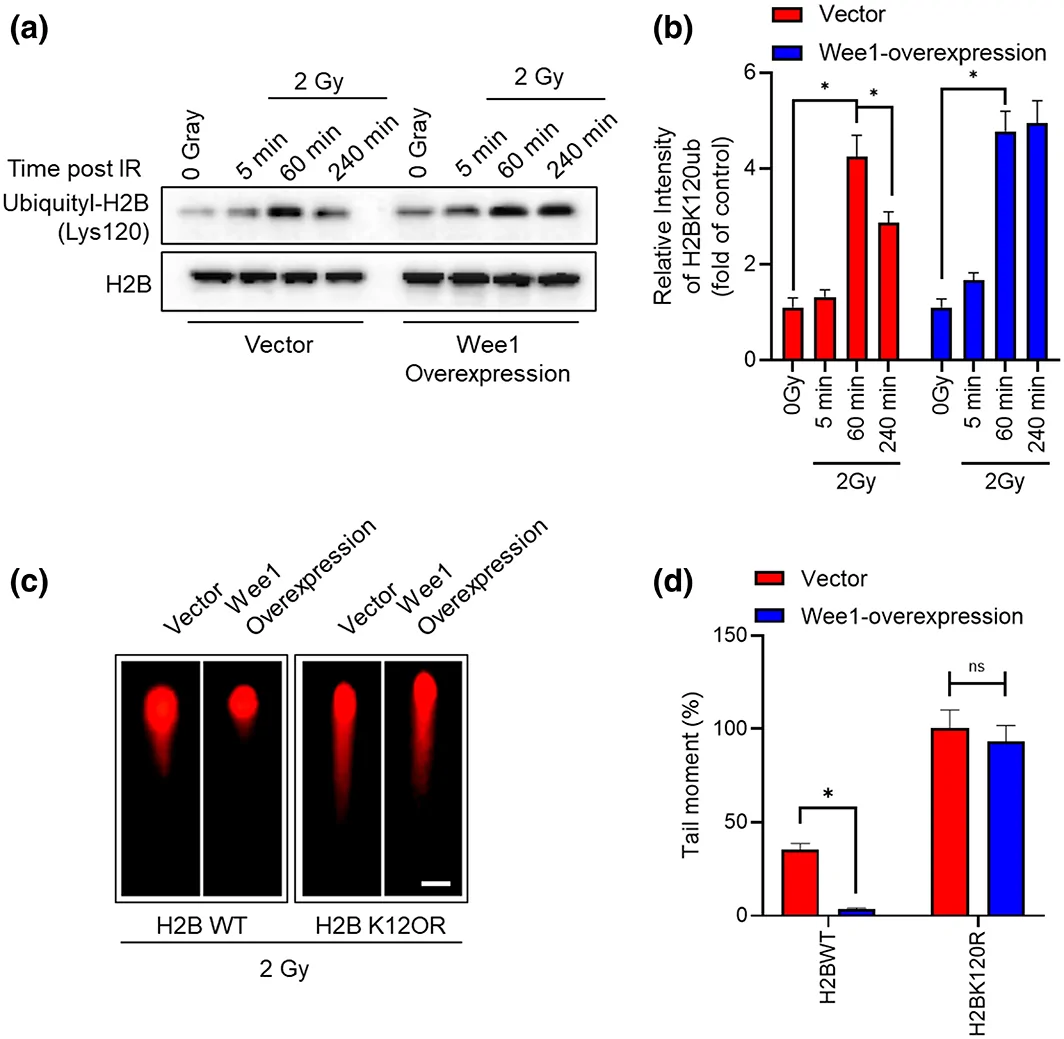
Overexpression of Wee1 increases the level of H2BK120ub and alleviates ionizing radiation (IR)‐induced DNA damage in small‐cell lung cancer cells. (a) Western blot results show the expression of H2BK120ub at different time points post‐IR in wild type (WT) or Wee1‐overexpression DMS114 cells. (b) The statistical analysis of the relative intensity of H2BK120ub level. (c) Comet assays show the DNA damage results post‐IR in H2B WT or H2BK120R group after Wee1 overexpression. (d) Relative tail moments statistics. Data are presented as mean ± SD for at least three independent experiments. Asterisks indicate statistical significance (*p* < 0.05).

The dynamic change of H2B mono‐ubiquitination at lysine 20 might be involved in IR‐ and drug‐induced DSB in SCLC cells. To evaluate the role of Wee1 in H2BK120ub and to identify the appropriate time point of Wee1 in H2BK120ub after DSB in SCLC cells, the Wee1‐overexpressed plasmid was transfected into DSM114 cells, and IR induced the DSB. As illustrated in Figure 1a, the level of H2BK120ub was evaluated by Western blot assay at different time points (5, 60, and 240 min). It was found that IR could induce an apparent increase in H2BK120ub in the DMS114 cell line in both WT‐ and Wee1‐overexpression groups. In addition, as illustrated in the statistical analysis (Figure 1b), overexpression of Wee1 significantly prolongs the dimension time of H2BK120ub under IR. The comet assay results indicated that the level of DNA damage was significantly decreased in the Wee1‐overexpression group in H2B WT DMS114 cells. Interestingly, in H2BK120R DMS114 cells, overexpression of Wee1 did not alter the DNA damage level. These results suggest that Wee1 could alleviate IR‐induced DNA damage in SCLC cells, which might be related with the ubiquitiniation modification of H2BK120.

### H2BK120ub is a crucial molecule in Wee1‐mediated DSB in SCLC cells

**FIGURE 2:**
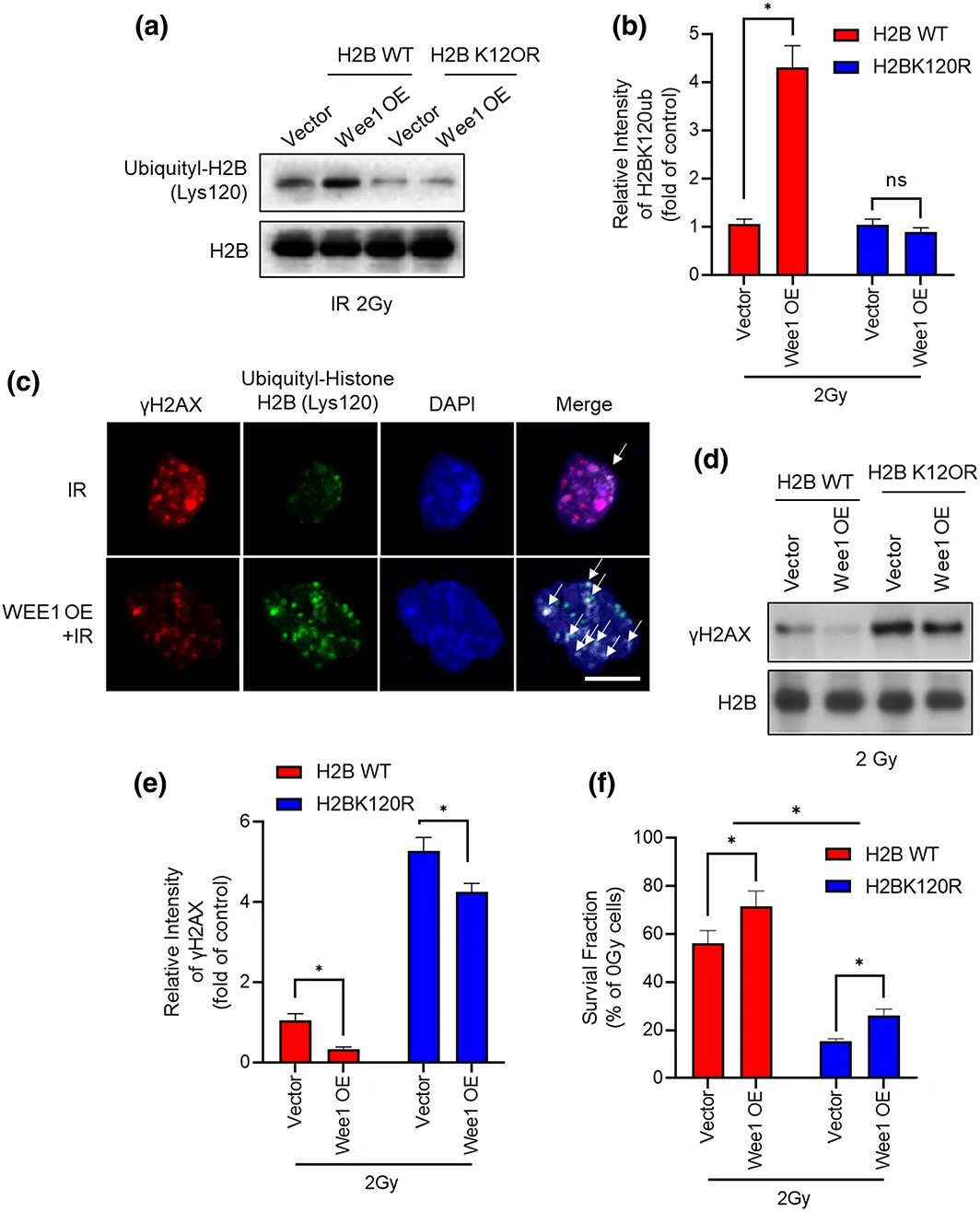
H2BK120ub is a crucial molecule in Wee1‐mediated double‐strain break repair in small‐cell lung cancer cells. (a) The effects of Wee1 on the expression level of H2BK120ub under ionizing radiation (IR) in WT or H2BK120R mutant DMS114 cells subtype. (b) The relative intensity of the expression level of H2BK120ub under IR in WT or H2BK120R mutant DMS114 cells subtype. (c) Immunofluorescence shows the effects of Wee1 on the colocalization of γH2AX and H2BK120ub post‐IR in DMS114 cells. (d) The expression of γH2AX was determined by Western blot assay in WT or H2BK120R mutant DMS114 cells subtype with or without Wee1 overexpression. (e) The relative intensity of (d). (f) Cell viability assay results illustrate DMS114 cell survival post‐IR (24 h) in WT or H2BK120R mutant DMS114 cells subtype, with or without Wee1 overexpression. Data are presented as mean ± SD for at least three independent experiments. Asterisks indicate statistical significance (*p* < 0.05).

To identify the specific role of H2BK120ub on Wee1‐mediated DNA DSB repair, we transfected the H2BK120R plasmid into the DMS114 cell line to simulate the deficiency of ubiquitination. The transfection efficiency was evaluated by Western blot assay (data are not shown). As illustrated in Figure 2a, after IR (2 Gy) treatment, the level of H2BK120ub significantly increased in the Wee1‐overexpression group in H2B WT DMS114 cells. However, in DMS114 cells with H2BK120R, no significant alteration was observed between the vector and Wee1‐overexpression group (Figure 2b). In addition, the colocalization of the phosphorylation of histone variant H2AX at serine 139 (known as γH2AX, a usual marker of DSB) was observed in Wee1‐overexpression DMS114 cells under IR treatment (Figure 2c,d). Moreover, γH2AX was potently downregulated after overexpression of Wee1 in H2B WT DMS114 cells under IR (Figure 2e). Interestingly, although a statistical decrease in γH2AX was observed in Wee1‐overexpression‐H2BK120R‐transfected DMS114 cells, the decline is not apparent (Figure 2f). Furthermore, in the cell viability assay results, although Wee1 overexpression significantly increased the survival rate of DMS114 cells after IR treatment, interestingly the cell viability significantly decreased in the H2BK120R‐transfected group compared to the WT group. These results suggest that H2BK120ub is a key molecule in Wee1‐mediated DSB repair in SCLC cells.

### H2BY37ph is involved in Wee1‐mediated H2BK120ub through E3 ubiquitin ligase

**FIGURE 3:**
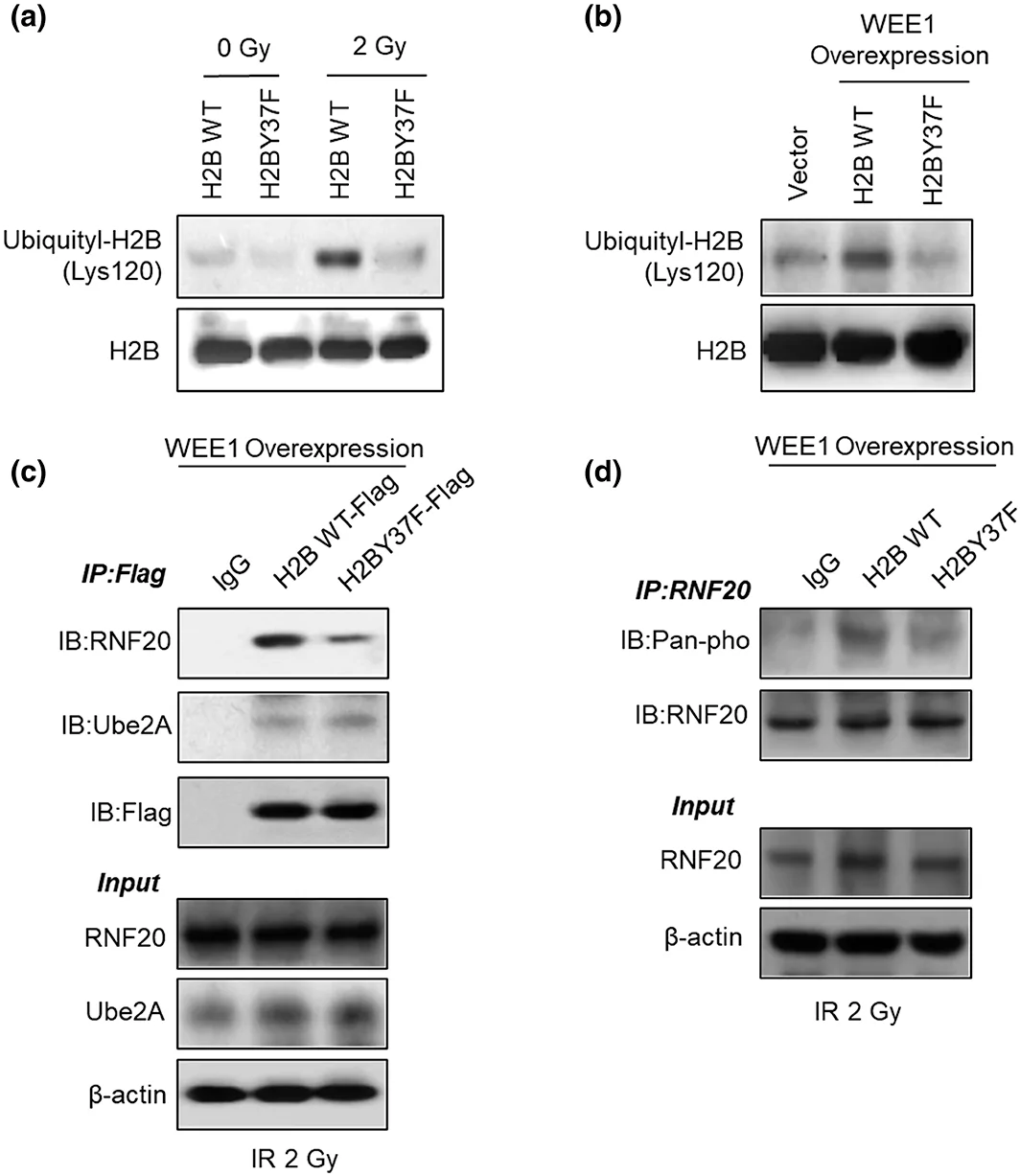
H2BY37ph is involved in Wee1‐mediated H2BK120ub through E3 ubiquitin ligase. (a and b) The effects of Wee1 on the expression level of H2BK120ub under ionizing radiation (IR) in WT or H2BY37F mutant DMS114 cells subtype. (c) Co‐immunoprecipitation shows the interaction of H2B with RNF20 or Ube2A in WT or H2BY37F mutant DMS114 cells subtype post‐IR. (d) Determination of RNF20 phosphorylation alteration mediated by Wee1 and H2BY37F mutant DMS114 cells subtype post‐IR. Data are presented for at least three independent experiments.

H2BY37ph is an identified substrate of Wee1. It is therefore speculated that Wee1‐mediated H2BK120ub might crosstalk with H2BY37ph. As illustrated in Figure 3a, after treatment of DMS114 cells with IR, the level of H2BK120ub significantly downregulated in the H2BY37F‐transfected DMS114 cell line (Figure 3a). Similarly, in the Wee1‐overexpression group, the ubiquitination on H2BK120 significantly decreased compared to the H2B WT group (Figure 3b). As the ubiquitination of H2B is dependent on E2 (Ube2A) and E3 (RNF20/40) ubiquitin ligase, we conducted the co‐immunoprecipitation assay to determine the potential interaction between H2BY37ph and E2 or E3 ubiquitin ligase. As illustrated in Figure 3c, the interaction of RNF20 with H2B (H2B WT group) significantly increased after IR treatment in Wee1‐overexpression DMS114 cells. However, in the H2BY37F group, such interaction was significantly weakened. Interestingly, no significant alteration was observed for the E2 ubiquitin ligase Ube2A between the H2B WT and H2BY37F groups (Figure 3c). In addition, it is observed that the phosphorylation of RNF20 was significantly upregulated, while in the H2BY37F group, the RNF20 phosphorylation level potently decreased compared to the H2B WT group after IR treatment in the Wee1‐overexpression DMS114 cells (Figure 3d). These results suggest that there is crosstalk between H2BY37ph and H2BK120ub in Wee1‐overexpression SCLC cells after IR treatment in an E3 ubiquitin ligase‐dependent manner.

### H2BY37ph regulates Wee1‐mediated IR‐induced DSB repair

**FIGURE 4:**
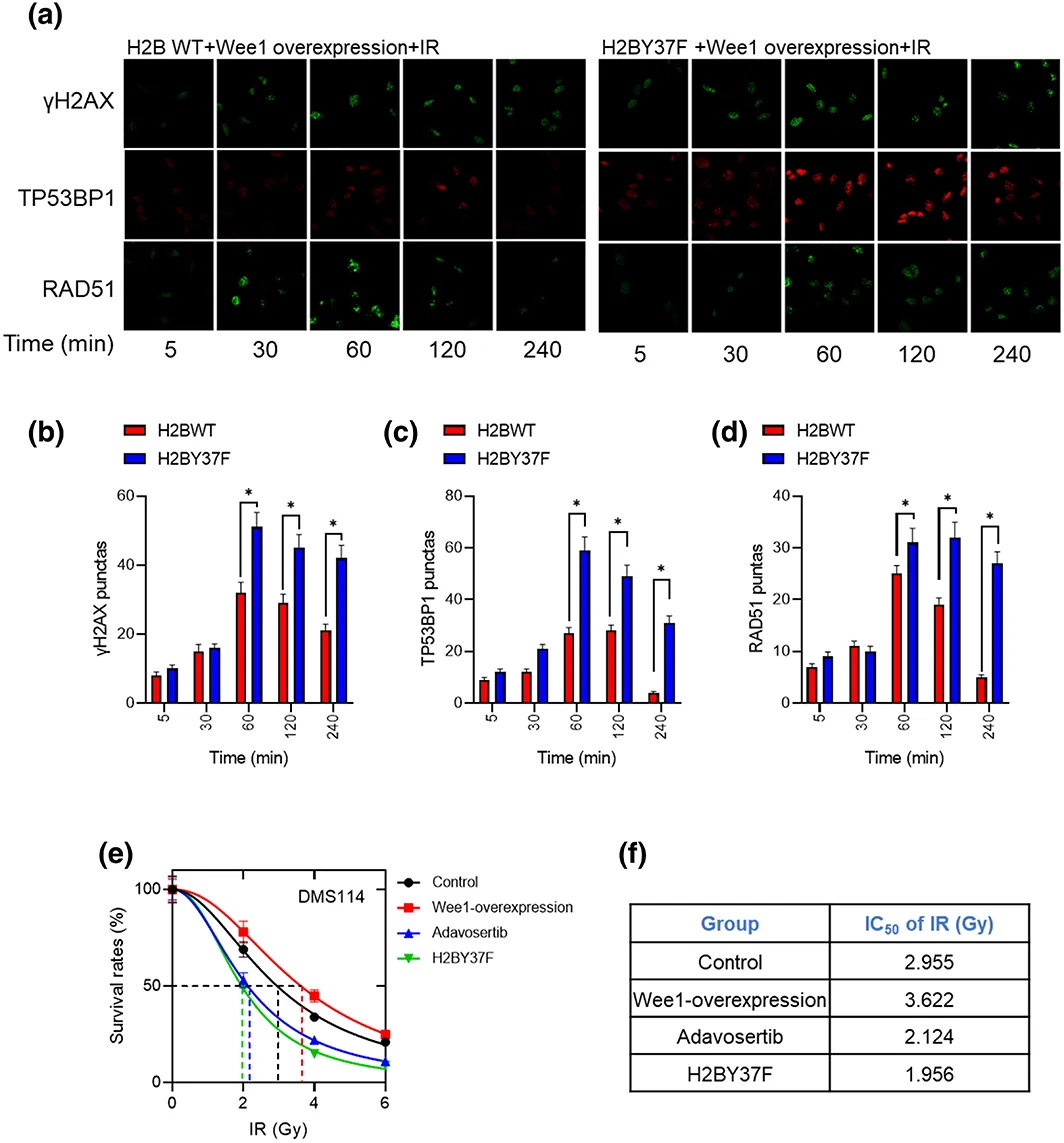
H2BY37ph regulates Wee1‐mediated ionizing radiation (IR)‐induced double‐strain break repair. (a) The foci of γH2AX, TP53BP1, and RAD51 were determined by Immunofluorescence in WT or H2BY37F mutant Wee1‐overexpression DMS114 cells subtype post‐IR at different time points. (b–d) The statistical analysis of (a). (e and f) The cell viability of different H2B subtype DMS114 cells (Wee1 overexpression) post‐IR (24 h). Data are presented as mean ± SD for at least three independent experiments. Asterisks indicate statistical significance (*p* < 0.05). [Correction added on 9 May 2023, after first online publication: In figure 4, label a, one misused picture was corrected. Also, ‘IC50 of IR’ has been updated to ‘IC_50_ of IR (Gy)’ in label f.]

To determine the effects of H2BY37ph on Wee1‐mediated DSB repair, we evaluated the dynamic change of γH2AX, TP53BP1, and RAD51 alteration under IR in Wee1‐overexpression DMS114 cells. As illustrated in Figure 4a, in the H2B WT group, the foci of nuclear γH2AX, TP53BP1, and RAD51, significantly increased after IR treatment at different time points. Interestingly, in the H2BY37F group, the dismission of relative focis of nuclear γH2AX, TP53BP1, and RAD51 was significantly slower than in the H2B WT group (Figure 4b–d). Moreover, in the cell viability results, DMS114 cells transfected with H2BY37F plasmid illustrated a higher sensibility towards IR treatment and the IC_50_ value in the H2BY37F group was significantly lower than that in the control, Wee1‐overexpression, or adavosertib (Wee1 inhibitor) groups (Figure 4e,f). These results suggest that H2BY37ph could regulate Wee1‐mediated DSB repair in SCLC cells.

## DISCUSSION

As the Achilles' heel in cancer treatment, DDR is one of the crucial mechanisms utilized by a series of therapeutic strategies, including chemotherapy (cisplatin), targeted therapy, and IR treatment.^8^ For SCLC management, systemic chemotherapy, typically cisplatin combined with etoposide, is the preferred initial treatment regimen in the early stages of SCLC diagnosis in patients with limited and extensive disease, and may be combined with surgery, local radiotherapy or immunotherapy, depending on the stage of disease progression. Reassuringly, the response rate of cisplatin/etoposide in SCLC patients exceeded 60% in the early phase of tretment.^9^, ^10^ Regrettably, most SCLC patients experience relapse and resistance within 6 months of initial treatment, and median survival after developing resistance to such therapy is only 4–5 months, resulting in generally low long‐term survival rates for SCLC patients. Cisplatin binds covalently to tumor cell DNA and denatures it to form intra‐strand cross‐links, damaging DNA. In addition, etoposide blocks topoisomerases from detaching from DNA in tumor cells, leading to DNA double‐strand damage. More typically, IR attacks DNA and produces a variety of DNA lesions, mainly including DNA DSBs.^11^, ^12^, ^13^ DNA damage caused by these methods can induce tumor cell growth arrest and apoptosis by blocking DNA transcription and replication functions,^14^ therefore DDR is a determining factor in the sensitivity of these treatment options. Tumor cells can progressively evade and counteract the therapeutic effects of platinum‐based chemotherapy or radiotherapy by means of DDR mechanisms. In‐depth investigations into the mechanism of DDR in SCLC are important for the improvement of the survival of SCLC patients.

In the present study, we determined the potential mechanisms of action on Wee1‐mediated DSB repair in SCLC cells, which is dependent on the H2BK120ub. Moreover, we found that the substrate of Wee1‐H2BY37ph is crosstalk with H2BK120ub through interacting with E3 ubiquitin ligase (RNF20/40) and upregulating its phosphorylation level, which plays a critical role in Wee1‐mediated DSB repair.

DNA damage activates many signaling pathways that regulate the DDR, persistently blocking cells in the G2/M phase and eventually activating cell cycle checkpoints during which DNA repair is completed, thus resisting mitotic catastrophe and cell death caused by excess DSB.^15^ The regulatory factors of tumor cell cycle arrest induced by chemotherapeutic agents or radiotherapy have long attracted extensive attention.^16^ It has been found that cisplatin sensitivity in yeast is associated with cell cycle arrest induced by Wee1, an intranuclear tyrosine kinase.^17^ Our previous results showed that the degree of drug resistance in SCLC cell lines was proportional to both the transcription and expression levels of Wee1, suggesting that Wee1, an evolutionarily highly conserved kinase, plays a vital role in the therapeutic resistance of SCLC.^18^

However, there are conflicting views on the mechanisms by which Wee1 regulates tumor resistance. It is well established that Wee1 is an important cell cycle regulatory signal in tumor cells, and its role in regulating DDR and genomic stability is widely acceptable.^19^ A study in breast cancer found that inhibition of Wee1 expression could target and correct cell cycle checkpoint abnormalities, thereby overcoming tumor cell resistance to cisplatin.^20^ Moreover, some studies have revealed through clinical trials the great potential of Wee1 inhibitors in combination with poly ADP‐ribose polymerase inhibitors as resistance to first‐line therapy in clinical SCLC.^21^ Thus, although the exact mechanism of action of Wee1 in SCLC therapeutic resistance has not been conclusively established, given the direct association of SCLC therapeutic resistance with cell cycle arrest due to DNA damage, the regulation of SCLC drug resistance by Wee1 through cell cycle checkpoints is currently considered as the classical pathway for its action. However, as a protein kinase in the nucleus, the regulatory role of Wee1 in tumor cells is not limited to the regulation of cell cycle checkpoints. It has been found that Wee1 is a key factor in the regulation of histone synthesis, suggesting an essential role for Wee1 in epigenetic aspects.^22^ A study on NSCLC found that Wee1 interacted with miRNAs, which in turn caused drug resistance and accelerated the disease process.^23^ Thus, there may be a nonclassical pathway of Wee1 that does not lead to drug resistance in SCLC through cell cycle checkpoints, but whether this type of mechanism of action is real and even whether it can serve as a target for antagonizing drug resistance in SCLC are key questions that need to be addressed.

Studies have shown that H2BK120ub is a regulatory axis for DNA DSB signaling and repair, which could cause elevated levels of H2Bub without affecting posttranslational modifications of H3K4me3, H3K79me2, and other histones associated with gene transcriptional regulation.^24^ Hence, it is speculated that ubiquitination of H2B might be involved in Wee1‐mediated DSB repair, which is a crucial mechanism for therapy resistance. We found in the present study that mono‐ubiquitination of H2BK120 plays an essential role in Wee1‐mediated DDR in SCLC cells. We first determined the H2BK120ub level at different time point post‐IR in SCLC cells in the Wee1‐overexpression group. The results indicated that overexpression of Wee1 significantly induced increasing H2BK120ub, suggesting that H2BK120ub may be involved in Wee1‐mediated DSB repair. We also conducted the comet assay, an Immunofluorescence technology that could reveal the extent of the DNA damage level. The results indicated that Wee1 overexpression attenuated DNA damage levels in wild‐type H2B SCLC cells. Interestingly, Wee1 overexpression failed to rescue the DNA damage degree in H2BK120R‐transfected SCLC cells under IR. These novel findings indicate that mono‐ubiquitination of H2BK120 may act as a critical molecule on Wee1‐mediated DNA damage under DSB. There is no noticeable increase in the H2BK120R group under IR even at a Wee1‐overexpression condition. Moreover, upregulation of co‐localization of H2BK120ub and γH2AX was observed in the Wee1‐overexpression group under IR in SCLC cells. In addition, H2BK120R‐transfected SCLC cells are more sensitive to IR, indicating that H2BK120ub is a path for Wee1‐mediated DSB repair.

H2BK120ub at different locations in the genome can crosstalk with other types of histone modifications to regulate gene transcription.^25^, ^26^ In addition, DNA DSBs induce H2BK120ub enrichment, and the H2Bub in this process is not crosstalk with gene transcription‐related modifications such as H3K4me3 and H3K79me2, which only provide spatial site block and support for DDR and DNA deconvolution, and the transition between ubiquitination and acetylation at the H2BK120 site becomes a critical turning point in response to DNA damage and initiation of the HR repair pathway, suggesting that there is likely an epigenetic regulatory mechanism for H2Bub in regulating DSB repair that is not dependent on gene expression.^24^, ^27^ It was shown that structural changes in the N‐terminal of H2B or site‐block changes in the core have the potential to allow greater exposure of the C‐terminus and thus provide modification potential,^27^ while phosphorylation or conformational changes in the core regions of histones H2A and H3 have similar functions.^28^, ^29^ In addition, in both yeast and mammalian cells, Wee1 binds and catalyzes histone H2BY37ph and abnormal expression of Wee1 causes aberrant phosphorylation modification of the H2BY37 locus, which in turn affects intranuclear protein expression including isocitrate dehydrogenase 2.^30^, ^31^ Furthermore, H2BY37ph is focally clustered at the histone cluster gene loci and there is a high cell cycle association.^32^ All the above evidence suggests that H2BY37ph may be involved in Wee1‐mediated treatment resistance in SCLC. In the present study, we noticed that H2BY37F could prevent the increase of the H2BK120ub level in Wee1‐overexpression level at both normal and Wee1‐overexpression conditions, indicating that H2BY37ph crosstalk with Wee1‐mediated H2BK120ub.

The ubiquitination of the H2B enzymatic cascade involves E3 ubiquitin ligase, which is mainly accepted to be the RNF20‐RNF40 complex.^33^ In addition, the ubiquitin‐conjugating enzyme 2A (UBE2A) is also generally known as the chief E2 ubiquitin ligase involved in the ubiquitination of H2B, which also functions as the E2 ubiquitin ligase for the nuclear antigen that activates in DNA repair.^34^ Hence, we detected whether H2BY37ph could recruit these two molecules to regulate H2BK120ub. The results indicate that H2BY37ph has a significant interaction with the RNF20‐RNF40 complex, but not UBE2A, indicating that H2BY37ph crosstalk with H2BK120ub through E3 ubiquitin ligase. Moreover, it is documented that the phosphorylation of RNF20 induced by ataxia‐telangiectasia mutated proteins (ATM) is a critical pathway for ubiquitination of H2B in DSB repair. We therefore detected whether H2BY37ph could impact on the phosphorylation of RNF20.^35^ The results show that mutation of the phosphorylation site at H2BY37 significantly decreases the phosphorylation level of RNF20, indicating the significant role of H2BY37 on Wee1‐mediated H2Bubi.

In conclusion, high expression of the key regulatory factor‐Wee1 and further catalyzing H2BY37ph, which produces a crosstalk between H2BY37ph and H2BK120, leading to elevated levels of H2BK120ub that in turn promote DSB repair, resist the mitotic catastrophe otherwise caused by excess DSB, and ultimately lead to treatment resistance in SCLC. The mechanism is mainly that H2BY37ph recruits E3 ubiquitin ligase and promotes its phosphorylation level upregulation. This study clarifies the nonclassical mechanism of Wee1 regulation of SCLC therapeutic resistance, which provides a theoretical basis for the clinical understanding of the regulatory network of Wee1 and its use as a target for overcoming therapeutic resistance.

## AUTHOR CONTRIBUTIONS

All authors have contributed significantly, and all authors read and approved the final manuscript.

## FUNDING INFORMATION

This work was supported by Tianjin Education Commission Scientific Research Project (2019KJ183).

## CONFLICT OF INTEREST STATEMENT

The authors declare that the research was conducted in the absence of any commercial or financial relationships that could be construed as a potential conflict of interest.

## Data Availability

Data are available upon request to the correspondence author.
